# Environmental racialisation and poetics of influence in the postgenomic era: fire, soil, spirit

**DOI:** 10.1136/medhum-2020-012061

**Published:** 2021-04-20

**Authors:** Lara Choksey

**Affiliations:** Wellcome Centre for Cultures and Environments of Health, University of Exeter, Exeter, UK

**Keywords:** cultural studies, genetics, metaphor, poetry, medical ethics/bioethics

## Abstract

This article considers processes of environmental racialisation in the postgenomic era through their politics of difference and poetics of influence. Subfields like epigenetics promise to account for a plurality of possible influences on health outcomes. While this appears to present possibilities for historical reparation to communities whose epigenomes may have been chronically altered by histories of violence and trauma, the prevailing trend has been to compound processes of racialisation in the reproduction of good/bad environments. The postgenomic era has promised an epistemological transformation of ideas and values of human life, but its practices, technologies and ideology have so far prevented this. Epigenetics, rather, reproduces biomedical exclusions through imaginaries of embodied contexts, methods of occlusion and hypervisibility, and assignations of delay and deviance. This is more complex than both genetic reductionism and environmental racism: studies on epigenetics reveal a poetics of influence at work under liberal humanism complicit in the creation of death-worlds for racialised populations. Other experiments with life are possible and unfolding: Jay Bernard’s poem ‘Chemical’, set in the aftermath of London’s Grenfell Tower fire in 2017, unmoors its bodies from material environment, offering a spectral configuration of collective life. This configuration involves negotiating with the fixing of time and space on which genomic imaginaries depend.

A complex negotiation is unfolding in the postgenomic era that moves unevenly and unpredictably across the politics, practices and forms of genetics in society. This negotiation concerns the stability of biological categories and the influence of environmental relations, or between what is understood as an organism, the multiplicity of relations that make up what is called its environment, and how the organism comes to be through and with these environments. This article considers poetics of influence in the postgenomic era: how the body is imagined and produced as an object of knowledge, and how this forecloses what can be said or supposed about the relations through which it unfolds in and with its surroundings. To do this, I look at three imaginaries of influence operating in epigenetic research that extend across conceptions of space, methods of data use and figures of time: embodied context (space), occlusion and hypervisibility (method), and delay and deviance (time, or bodies that waste time). Taken together, these imaginaries exceed the framework of the biopolitical; instead, they constitute a necropolitical process of environmental racialisation, which I understand here as the ongoing creation of what Achille Mbembe calls death-worlds: ‘new and unique forms of social existence in which vast populations are subjected to conditions of life conferring on them the status of living dead’.1 In epigenetic contexts, this means determining and fixing bodies within context, method and time, through techniques that continue to produce biomedical value in postgenomic contexts.

Addressing the uneasy and often obscure poetics of influence that operate to construct the relations between an experimental object and its environments is necessary for thinking through the possibilities for collective practices of health. This means rethinking the body as molecular object, the environment as static and extractable spatiotemporal unit, as well as the classifications that straddle both object and influence. Epigenetics—the study of non-genetic influences on genetic expression throughout an organism’s lifecourse—has at turns permitted, encouraged and forced confrontations between the life sciences, the social sciences and the humanities because of the possibilities it opens up for reconceptualising social relations, human and non-human interactions, and the influence of human history on biological heredity. If an individual’s genetic programme is not fixed at conception, if the organism is subject to any number of epigenetic events throughout its lifecourse that might alter the genes it expresses, and if the variables of environmental stimuli might range from smoking to maternal care, to weather, to the genomes of the organisms our bodies are populated by, then what happens to the stability of ‘the organism’ as an object of knowledge: sociohistorical, political, material?

To Alfred North Whitehead, the organism appears ‘in its completeness’ as an invention of industrial capitalism:

A factory, with its machinery, its community of operatives, its social service to the general population, its dependence on organising and designing genius, its potentialities as a source of wealth to the holders of its stock is an organism exhibiting a variety of vivid values.2

Figured as an organising principle and generative idea of a phantasmagoria of attachments, Whitehead’s organism, here, is a site of mechanical reproduction. As long as epigenetics remains within the terms of post-Enlightenment bioscience which relies on a conceptual distinction between organism and environment, or in Whitehead’s terms, subject and predicate, the life sciences will continue to produce the organism as a natural unit of production that is both situated *in* and productive *of* socioeconomic relations.

Unwriting the poetics of influence that construct the organism in this way—as both discrete and replaceable, productive and expendable—is not only a project of antireductionism. While the reparative potential of epigenetics might loom large in marginalised health contexts, racial capitalism continues to engender practices of classification for ordering the world into a typology of surplus value, and to structure methods of interpretation within the life sciences. Of primary interest to Big Bio is making the matter of life an object of exchange, and this involves telling certain stories about biomedical futures to those who are able to buy into them. Jenny Reardon has urged deeper investigation into the political economy of the postgenomic era and the transformation of Euro-American liberal democracies into economies of information.3 Countering quick-fix alternatives to the deep epistemological overhaul required in the postgenomic moment requires more than fortifying liberal diversity mechanisms of inclusion/exclusion (ie, gathering more data, the better to interpret marginalised health profiles and to tackle health disparities along the lines of marginalised identifications). In this situation, Reardon notes that ‘attention to poetics may matter more than principles’.4 Transforming the epistemological footholds of the postgenomic era requires rethinking its poetics of life, insofar as language has made possible the molecular reduction of the former, and speculative and indefinite holding-at-bay of the latter.5 This project also means confronting the organisation of biomedical industries around forms of value that naturalise social articulations of difference. The continued naturalisation of difference across and within species extends across spatial, methodological and temporal configurations, within a particular metaphorical infrastructure of extraction, profit, expendability and destruction.

In the final section of this article, I turn to the state enquiry into soil contamination at the site of the 2017 Grenfell Tower fire in West London. I read the process of this enquiry through the lens of environmental racialisation, in which the remaining residents were fixed to toxic ground of the site while forced to take responsibility for knowing the land in their campaign for a thorough state investigation into the long-term environmental effects of the fire. Alongside this, I read Jay Bernard’s poem ‘Chemical’ from the 2019 collection, *Surge*, which offers a speculative poetics that intervenes in the reproduction of Grenfell as a death-world by attending to its ‘spirit-level’, suspending a ‘with’ between the living and the dead. Through this, Bernard makes other descriptions and forms of surrounding relevant, soliciting a counterpoetics for the postgenomic era more interested in the messiness of entanglements between the living and the dead, as well as their endurance.

## Epigenetic poetics of influence

In this section, I consider the question of influence in epigenetic research, and how epigenetics’ interdisciplinary reach across the humanities and social sciences indicates both an established resistance to a lineage model of influence in fields outside the life sciences, as well as the construction of these alternative accounts of influence in literature. At the same time, theories of culture often assume a selective model of influence whereby certain cultural traits and forms are propagated for reasons both formal and historical: as Isabelle Stengers puts it, ‘the omnipotence of selective sorting’,6 or in Raymond Williams’s words, the ‘regular slide towards a past tense and fixed form’.7 Given its concern with methods of interpretation, epigenetics might figure as a transdisciplinary analogy for current pressures on literary studies to think through the ways that genealogies of literary and cultural influence are formulated and formalised. Epigenetics, in this light, operates as both analogy for and agent of ‘changes in structures of feeling’,8 and here I foreground its role in racialisation as a process of social experience.

The scale of the philosophical challenge that the postgenomic era has set out to liberal humanism has been indicated in different ways in recent work by Wendy Wheeler, Catherine Malabou, Clare Hanson and the collective school of biosemiotics.9 To different degrees these studies consider epigenetics as a radical overturning—or, in Malabou’s case, implicit tendency—of post-Enlightenment metaphysics. A decolonial approach would be to consider the postgenomic moment to be as regional to Eurocentric knowledge systems as the Human Genome Project was. Genomics is not a universal ontology, but a regional manifestation of a particular worldview that has become globalised and whose globalisation has been facilitated by violent processes of extraction. However commonplace the conceptual models and technologies of the Human Genome Project became, its premise was banked in a transhumanist genealogy of transcending human limitations, whereby the function of social is to provide environments for superior humans to emerge and live longer, fitter lives.

Since the first draft sequence of the human genome was published in 2001, a complex negotiation between body and environment has unfolded, fragmented across a number of sites of enquiry and modes of address. The once-peripheral field of epigenetics has moved closer to the centre of genomics’ speculative futures for human health. This movement unfolded in stages: first, the realisation that the number of protein-coding genes that humans express is roughly equivalent to *Caenorhabditis elegans*, a type of worm. Second, unlike *C. elegans*, which expresses almost all of its genetic material, humans express a tiny percentage. There is a comparatively vast amount of room for manoeuvre within what had been called junk or non-coding DNA, which goes some way to accounting for the differences in complexity between worms and humans. Third, that non-coding DNA can be expressed or silenced (methylated) according to various stimuli both internal and external to the organism. As many critics have argued, and as I go on to explore, these inferences have big implications not only for applications of scientific knowledge in biomedicine, but also for placing pressure on the philosophical underpinnings of the modern life sciences.

Up until the sequencing of the human genome, genetic heredity had been (largely) figured with a linear model of influence that bore close resemblance to a production line. Classical genetics understood genes as immutable hereditary material passed from generation to generation. After the discovery of the double helix in 1953, this model was figured in Francis Crick’s Central Dogma as the unidirectional transcription of DNA into RNA, followed by the translation of RNA into protein. As a model of influence, the Central Dogma also upheld the clockwork time of industrial capitalism, which E. P. Thompson renders as a reversal: from time measured by the time it takes to complete a particular task in peasant societies to ‘labour timed by the clock’ for ensuring mechanical synchronicities on factory floors.10 The linear model of genetic reproduction has also promoted the individualism of particular lineages, seeming to offer a genetic syntax for identifying ‘good stock’ at a molecular level. These are not the utopian fantasies of interwar eugenicists: Big Bio now purports to offer the means to edit parts of the human genome and to model future citizens for postindustrial futures.

However, the dynamics of the relations between organism and environment remain a subject of considerable uncertainty, while these relations continue to be framed as both divisible and calculable. For some, epigenetics offers a paradigm shift not only for understanding the processes but also in determining the politics of life itself, its arrangements and attachments. Wheeler argues that epigenetics moves us from the informational model of the biosciences that has dominated molecular biology since the 1950s, to a relational ontology in which ‘all life everywhere is about communication, semiosis, interpretation and meaning-making’.11 This departs from the coloniality of a world ‘out there’ to be processed by increasingly complex and capitalised-upon mechanical interventions. While this might sound like a biophilosophy of hermeneutics, something different is at stake here: this is not an endless play of signs in imagining possibilities for adaptation, but a project to establish the process by which certain meanings and their effects become possible. Staying with poetics does not mean suspending the hermeneutic as much as attending to the generation of interpretative gestures that reproduce necropolitical governmentality, and the imaginative registers in which these gestures become constituted as biomedical grammars.

There have been calls to be strategic with the genre of essentialism that epigenetics introduces, particularly around its reparative implications as evidence for wide-scale trauma in the wake of crimes against humanity. In the context of Indigenous Australian reparations, Megan Warin, Emma Kowal and Maurizio Meloni identify possible forms of ‘biolegitimacy’ in which ‘the epigenetic body is used as an historical testimony of colonial violence’.12 Adapting Gayatri Chakravorty Spivak’s postcolonial injunction, they call this ‘strategic biological essentialism’, mooted in a biopolitical economy of hope.13 Where Spivak describes the tactic of mobilising identity for the sake of shared objectives14—that is, temporarily suspending plurality for the sake of political representation—Warin, Kowal and Meloni suggest instrumentalising epigenetic proof of historical trauma as a way of gathering and emboldening Indigenous claims for reparation to settler colonial states. This is part of a longer wave of similar articulations around the politics of the (epi)genetic and historical trauma: among them, Thomas Grim and Clarence Wilson’s 1990 study on higher rates of hypertension among African American communities as an inheritance of conditions along the Middle Passage,15 and studies on the epigenetics of transgenerational trauma among the offspring of genocide survivors.16 These are situations marked as much by the absence of witnesses and the disappearance of evidence, and the molecular takes on a yet more rarefied status as a site where both the cause and effect of historical crimes can be tracked.

The question turns to what it is possible to know about influence in the postgenomic era: trajectory, memory and temporality. The contentions of epigenetics might resemble the belated arrival in the life sciences of a hermeneutic anxiety, but they also gesture to the necessity of working more slowly to derive some sense of what is at stake in the negotiation between, and identification of, different biomedical actors (genome, chemical, element), and in practices of differentiation. In these uneasy, uneven, and sometimes impossible articulations of influence, biomedicine does not have the resources to deal with the crisis of meaning engendered by the postgenomic. Barbara Prainsack writes:

In many instances, such as genetic analysis, or imaging, technology use has already ceased to be the bottleneck – interpretation is. Although much wider strata of people will be able to afford a genome sequence or a proteome profile in the near future, very few people would be able to afford the interpretation of this information.17

In the absence of reliable interpretations, discredited biological categories are regurgitated as placeholders for precision, which keeps them in circulation. Angela Saini addresses the residual role race and ethnicity play in biomedical research, in the context of biologised accounts of the disparities in COVID-19 death rates between black and Asian communities in the UK, and black and Latino communities in the USA. One of the reasons for the residual power of the race concept long past its expulsion from genetic explanations is, Saini suggests, because of the lure of personalised medicine ‘so precise that everyone’s biological profile is perfectly understood’, working on the basis of averaging shared health traits among a preselected group.18 These categories are reiterated as biological profiles in lieu of ‘complete’ personalisation. In countering the inability to interpret personalised health data, health researchers fall back on older interpretations of human difference, with race and ethnicity often at the foreground.

Josie Gill has offered a way of thinking race as a biofiction that is produced in language and transmitted into scientific praxis, ‘a social construct with biological consequences’.19 For Gill, the writing of racist environments in literary texts not only *represents* the tenacity of biological accounts of race, but are also interventions in their metaphorical infrastructure: ‘race is *created* at the intersection of the imagined and the real, through a focus on the impact of racist environments on the body’.20 This is not to offer a new theory of race via epigenetics, nor to suggest that any kind of stable meaning of race emerges from the physical and genealogical impacts of racism. Gill foregrounds instead the circulation of influence in processes of racialisation: how language creates environments that might alternatively (and simultaneously) realise, relieve and resist processes of racialisation. In the following section, I consider the poetics of embodying contexts in epigenetic research, before considering examples of environmental racialisation.

## Embodied contexts

In deriving a sense of coherence for its experimental hypotheses, studies in epigenetics construct temporal and spatial imaginaries of influence in communicating how the organism is understood to be affected by its environment over time. Bodies are fixed in particular spatiotemporal arrangements, imagined to be *in* a particular place *at* a particular time. This restates the idea of the organism as a ‘continuous succession of instantaneous material configurations’,21 across three dimensions: place is both geographical and ancestral, and it extends across boundaries of body and environment. The epigenome takes place across genealogical time, in that it is fixed uncertainly in the contexts of parental, grandparental and ancestral genomes, which may or may not become relevant as a given context demands. It also takes place across geographical space in the form of possible environmental influences (the chemistry of the womb, atmospheric pollution, socioeconomic position). This three-dimensional fixing of the body in space is not derived from imaginaries of entanglement, but is rendered on an ambiguous graph of possible influence, one whose geometric precision obscures biomedical uncertainty. It is the concert of precision and uncertainty that makes this graph valuable: the *staging* of possible influences is a way of keeping faith in the research, and its indeterminacy becomes part of an affective repertoire between patient and doctor. This graphic does not suspend familiar categories of identity, but invokes the biological within the framework of the social before returning to molecularised conceptions of identity via calculations of possible influence.

Various longitudinal studies on human cohorts have provided categories of data through which to analyse epigenetic events. Given that humans share more genes with mice than with plants, there has also been a degree of extrapolation between findings from studies on mice and their implications for human lifeworlds. Frances Champagne’s study on the transgenerational effects of maternal care investigated the effects of maternal grooming on the development of baby mice: in one group, the mouse mother licked her pups, while in another litter this repeated act of physical care was withdrawn.22 The latter litter became more prone towards obesity and shorter lifespans, and the study purported to show the deleterious effects of bad mothering on child development, effects that might be inherited by subsequent generations.

Sarah Richardson notes the formulation of the maternal body in such studies as an ‘epigenetic vector’, which she defines as ‘an intensified space for the introduction of epigenetic perturbation in development’.23 The idea of a vector operates through an imaginary of graphic imprecision, a way of describing ‘forms of causality that are conduit-like rather than strictly cause-effect’,24 more of a *pointing towards* than *generative of*. This choice of formulation indicates what Stephanie Lloyd and Eugene Raikhel call the ‘situated uncertainty’ in which epigenetic research is lodged when it comes to equivocating between cause and effect, operating in multiple experimental constraints—‘technical difficulties in identifying noise from data’, ethical quandaries and publication timelines.25 This uncertainty produces mixed messages for expectant mothers:

On one hand, women are instructed to do all they can to prevent harm to their fetus. At the same time, an individual woman can do little to improve outcomes for her own offspring if they are trapped in the intergenerational epigenetic “feedforward cycle” hypothesised by DOHaD [Developmental Origins of Health and Disease] research.26

The mode of this uncertainty, in practice, means that women are assigned primary responsibility for ensuring a good environment for future generations, while also having ‘very little power to influence their own outcomes’.27 The maternal body is both environment, ‘background element, a medium for the fetus’, and ‘a ‘critical’ developmental context in which environmental exposures are amplified, cues are transmitted, and genes are programmed’.28 This uncertainty does not generate a politics of difference akin to Jörg Niewöhner’s formulation of the embedded body: an indeterminate subject that emerges, entangled, between somatic and material influences, ideologies, and scientific praxis. Such a politics would involve a commitment to what he calls ‘building thick significance’ in interpreting between, for instance, ‘the wider biology of the case at hand … the mechanisms of gene expression … the experimental procedure [and] why certain instances of social change or perceptions of socio-economic difference may lead to certain somatic changes’.29 Rather, the maternal body-as-vector, writes Richardson, ‘*fixes* the molecular gaze *on* the embedded body, an already formed and highly-charged entity in the science of maternal-fetal relations, and elevates it to the centre of biomedical theory, intervention, and surveillance’.30 Sara Shostak and Margot Moinester extend this, arguing that this process of molecularising and individualising the environment ‘makes broader social structures and processes disappear’.31 Who is responsible for ensuring the good health of subsequent generations? Absent from many of these studies are larger-scale influences; instead, these influences appear as ephemeral suggestions in the margins of individualised trajectories split into discrete segments of experience.

The politics of difference in epigenetics makes bodies disappear into contexts. The fixed body is not figured as distributed across entangled and fluctuating channels of space-time, but fixed to a context in a calculation of difference embedded in placeholders of variable and invariable, subject and object, the done to and the doing, context and action. This is a process of disappearance because its tendency remains oriented towards the reductive, compressing plurality into individuated disaggregation, multitudes into correlated data, and removing the signs of wider systems in operation. It also makes invisible the kinds of influences that are not encompassed by technoscientific tabulations, and all the various and multiple ways in which beings are entangled with and through their surroundings. It is this difference, between ‘in’ and ‘with’, that characterises the political stakes of postgenomic poetics of influence, which in turn set the parameters and possibilities for taking responsibility for collective health.

Part of this process of disappearance involves the production of genomic norms; these might, in themselves, shift and mutate, but the point is to keep a value of standardisation locked into the graphic of human potential, standardisation here taking the place of imagining equitable collective systems. Becky Mansfield and Julie Guthman argue that epigenetics continues to operate on the genomic axis of ‘normal (inevitable) and abnormal (a sign of disruption)’: hence, ‘deciding what counters as improvement is embedded in normalisation’.32 For Mansfield and Guthman, epigenetics does not disprove but rather reproduces the normalisation of whiteness, or the positioning of whiteness as normative; it does so not through *genetic* determinism but *environmental* determinism, whereby racism becomes a causal factor in deviant and abnormal biological profiles. They write: ‘epigenetics offers a new form of racialisation based on processes of becoming’.33 Environment is ‘both *spatial* and *socionatural* – and is increasingly molecularised’, and it is also *temporal*: ‘epigenetic mechanisms crystallise – they make biological – the entire ‘environment’ in each specific place and time’.34 This inevitably leads to vocabularies of exposure and damage, and epigenetics becomes a way of studying ‘*disruption* to a normal, natural state of development – and variation represents defect and abnormality’.35 The logic moves from the racialisation of defect to the racialisation of defective influence: bodies exposed to bad environments, cultivated in faulty landscapes, become abnormal, biologically altered in deleterious ways by the lifestyles of themselves or their progenitors, by a plurality of elements in their surroundings, by exposure to disease, and so on. This offers a vocabulary for attaching bodies to abnormal, defective or inadequate environments, and for racialising their environments through imaginaries of bad influence.

This is not to suggest that epigenetics is simply an updated recourse to genetic determinism; something more sophisticated is going on here, which surpasses the parcelling of heredity into discrete units. Jasbir Puar's concept of ‘debility,’ a ‘triangulation of the ability/disability binary,’ apprehends the violence of this compounding of disability and environment into an imaginary of environmental disability: ‘The category of disability is instrumentalised by state discourses of inclusion not only to obscure forms of debility but also to actually produce debility and sustain its proliferation.’ The state recognition of individual disability, for Puar, ‘effac[es] the quotidian modalities of wide-scale debilitation so prevalent due to capitalist exploitation and imperialist expansion.’36 Following Puar, then, the production of embedded contexts introduces new categories of disability that obscure the superstructural production of debility through exploitative labour conditions, exposure to toxic environments, and reduced or minimal access to healthcare.

Epigenetics’s reliance on a multiplicity of possible influences, through a poetics of gesture that obscures its various reductions, generates a range of novel biomedical values to describe disability (individual, populational) while occluding systemic processes of racialised debilitation. This poetics of gesture points towards both to the possibility of a cure, and the importance of investing in continued technological innovation to pursue this possibility. It also extends the range of options for surveillance and tracking, not for the sake of interpretation, but to fix bodies to particular places by gesturing to the confluence of abnormality and bad environments. The imaginary of embedded contexts updates and extends a eugenic infrastructure for making disabled bodies disappear from the sociogenetic pool, for the sake of a future free from abnormality and deviance. In practical terms, the imprecision of epigenetic grammars of influence equates to forms of governance that are uninterested in empirical data fixed to the trajectory of individual development (supposedly the grail of epigenetics); instead, the body is figured as a data point *on which* possible influences may or may not have an effect.

## Epigenetic racialisation

While it promises a more holistic approach to mechanisms of genetic expression, the idea that bodies embody their contexts has meant retaining older categories of difference in deciding what influence affects which group. This is in part due to a deficit in causal explanations for particular epigenetic events. This does not just show up in the formulation of experimental parameters, but also in the ways that data are left out, occlusions that often reveal inconsistencies or oversights in collection practices; alternatively, data collection practices indicate the hypervisibility of race and ethnicity as biomedical categories of difference and the way that they often act as substitutes for personalisation. More broadly, the logic of embodied context fixes populations to their perceived influences—physiological, parental, chemical, cultural, regional or national—and fixes data differentiated by categories of race and ethnicity to these influences. In this section, I look at two examples of racialisation in epigenetics research, where race and ethnicity appear not in genetic markers, but through practices of self-identification and statements of exclusion. In both studies, race and ethnicity are imagined as causal factors that produce differences in data, while also attributed no specific genetic meaning. These categories function as floating signifiers, setting parameters for methods and analysis.37

The exclusion of data from non-Caucasian research participants reifies implicitly biological accounts of ethnicity-as-difference while giving no explicit biological explanation for what constitutes this difference. A recent study by Anne-Catherine Viuff et al on the effects of prenatal depression on fetal methylation patterns used data from the Bristol-based Avon Longitudinal Study of Parents and Children (ALSPAC). The Viuff study excluded data from non-Caucasians on the grounds that participation numbers were too low: the methods section accounts for this by saying ‘ALSPAC includes very few pregnancies of non-Caucasian ethnicity so we excluded those from the study’.38 Despite this exclusion, the study’s title neither acknowledges nor specifies the ethnic categorisation of its participants, only the source of its data. So how and where does ethnicity matter here? It is relevant enough to warrant the exclusion of ‘non-Caucasian’ data, but not relevant enough for its ethnic composition to be acknowledged in the title. This is an example of the confounding ways in which categories of ethnicity retain their purchase in studies that aim to show correlations between genetic expression, geographical ancestry and environmental influence, excluding non-Caucasian data while simultaneously framing Caucasian data as universal.

Epigenetics research upholds the mobilisation of racialisation on an axis of invisibility and hypervisibility: race and ethnicity are overdetermined categories of difference that are either awkwardly occluded or anxiously attended to. A 2017 paper on ‘differential methylation between ethnic sub-groups’ argues that this ‘reflects the effect of genetic ancestry and environmental exposures’.39 Rather than simply looking at the effects of smoking on methylation (one of the few epigenetic conclusions is that smoking unambiguously alters methylation patterns), the study is interested in the differences between different subgroups of Latino populations and in ‘the heterogeneity present within racial/ethnic groups and in admixed populations’. The study identifies a correlation between ‘ethnicity-associated sites’ and environmental exposure (in this case, the smoking maternal body as epigenetic vector). It concludes that a combination of ancestry and environmental factors ‘significantly contributes to variation in methylation’. That is to say, within the broad and—for the authors—problematically unspecific ‘Latino’ ethnicity group, there is significant variation in methylation patterns. But rather than departing from the logic of ethnicity, the study opts for an uneasy and empirically questionable compromise: the ‘sub-groups’ of Latinos were determined by the participants, who were given one of four prefigured options of identification: ‘Puerto Rican’, ‘Mexican’, ‘Mixed Latino’ and ‘Other Latino’. In this methodology, the process of ethnic identification remains, broken down into something slightly more plural but no less Eurocentric in its formulation of difference, and relies on the veracity and reliability of individuals’ knowledge about their own ancestry. Is the study interested in proving biosocial variation, or proving the epigenetic basis for ethnic categories?

Discussing the development of the drug BiDil—a heart medication marketed specifically to black consumers—Gill notes that the company’s use of self-reported identification as the basis for experimentation:

legitimised the use of racial categories in medicine and the idea that specific genetic markers could be linked to particular races, while simultaneously underscoring how fragile, unstable and incoherent race becomes in that context where it is accepted and worked with even though there was no justifiable medical or scientific grounds for doing so.40

The point demonstrated by all three examples is that race matters in imaginaries of biomedicine, even while its validity has no basis in genetic evidence. Occlusions, methods and conclusions like these remain possible because of the historical power of racialisation as a marker of biovalue, which here unfolds in a merging of ancestry, geography and undetermined environmental influences. The Galanter study is vague about what the ‘environmental factors’ at play in constituting the apparent biological difference between these subgroups might be, but the implication is that its participants’ historical formation into social units has created their biological difference—here figured as differential methylation patterns. Self-identification as a tactic of data capture becomes subsumed into a biologisation of ethnic and racialised difference.

These studies articulate the tenacity of racialised imaginaries in biomedical research on an axis of exclusion and overdetermination. At a very basic level, we might want to ask once more: *why is ethnicity being made to matter?* The study is an example of environmental racialisation, whereby ‘environmental differences’ contribute to descriptions of racialised difference. In Galanter’s study, this could easily be extrapolated into evidence of poor mothering among Latino women (smoking while pregnant); in Viuff’s study, self-reported depression among a largely middle-income, Caucasian group of women stands in for all women. Nor is it a coincidence that these studies are preoccupied with maternal responsibility, to follow Richardson. The environment of the home is central to the racialisation of environmental influence, and the bolstering of the normal/deviant model of heredity, expression and influence. These studies demonstrate methods of occlusion and overdetermination which make their subjects disappear into either the vagaries of racial and ethnic identifications, or universal maternal bodies. While their participants are ostensibly molecular storehouses of biopolitical calculation, these studies also depend on eugenic infrastructure that creates racialised death-worlds for those assigned as deviant, as I explore in the following section.

## Environmental racialisation

It is important to distinguish between practices that produce environmental racism and processes of environmental racialisation. While environmental racism has focused on unevenness of health disparities across lines of racial determination and geopolitical determinations of resource distribution,41 environmental racialisation embeds communities ‘in’ particular contexts in order to make quantitative and predictive assessments about their relative fitness, at the level of group or population. This is not biopolitical, but necropolitical; to follow Mbembe, this process upholds a racialised political economy that determines not only the right to administrate life, but the right to impose social or civil death.42 Discussing the organisation of race in Canadian cities, Cheryl Teelucksingh distinguishes environmental racism from environmental racialisation through the difference between ‘subjective intent’ and ‘unpurposeful racist outcomes’.43 I characterise the difference between these terms differently, given that the question of intentionality is a challenging starting position, which moves the focus away from institutionalised and systemic processes of colonial subjugation inscribed in law and social practices, which may or may not appear as intentional by individual actors. These codes and practices are often broken down into smaller units, so that comprehending the unity of their operation as racist is often difficult.

Starting instead with the assumption that colonial societies have well-developed and sophisticated processes for reconstructing the architecture of racism, it is important to begin with identifying how those processes unfold over time, and the imaginaries through which they are made to make common sense. Those who carry out these processes may or may not be fully cognisant of their implications; what is at stake, in the case of Galanter’s study on smoking and methylation, is how race is constructed through imaginaries of land, lineage and self-identification. More compelling is Teelucksingh’s argument that the concept of environmental racialisation allows a means of describing the social and spatial orientation of race.44 Thinking with Mansfield and Guthman, this argument can be extended to posit environmental racialisation as the reproduction of racialised difference through social, spatial and temporal orientations. This is tied to whom responsibility for arranging public life is assigned (state, family, maternal body, corporation, community), and to imaginaries of different developmental temporalities that certain groups are not only conceived to exist within, but to which they are assigned through formal and informal processes: housing policy, employment, leisure and recreational spaces, criminalisation, access to adequate healthcare, and—by no means least—global-national finance models that both uphold the uneven flow of resources away from the Global South, and sustain the North/South division as an environmental one of natural inequalities, rather than an economic one of extraction, labour division and resource distribution. Poverty becomes racialised context, imagined to produce biological difference.

Racialisation is produced through figures of delay and deviance. Mel Chen notes the temporal aspect of racialisation, characterised by delayed figures. They cite the example of ‘Mongoloid’ as an early name for Down’s syndrome, whereby ‘a non-white ‘race’ [is] used as a zone of deferral and marking, which accounts for the other kind of difference’45:

Within and away from the USA, the temporalized characterisations of delay have been variously applied to indigenous people and to racialised inheritors of histories of labour and enslavement – a broad swath that includes anyone less securely anchored to the leading edges of modernity.46

The logic of underdevelopment does not just appear in disparities in material infrastructure47; it is inscribed onto bodies in a naturalisation of racial and ethnic descriptions. Chen reads this as indicative of the continued influence of eugenics in biomedical discourses of enhancement and reproduction, as well as the ‘association of certain racial characteristics with cognitive deficiency’.48 Certain groups continue to be figured as living in delayed time zones, and this temporality is capacious enough to *extend between* geographical and ancestral markers, as well as to be *compounded by* ‘other environmental differences’. This delay functions as a grammatical premise for approaches to global, national, community and individual health research, where designated subjects ‘of European ancestry’ constitute normative genomic profiles while the data of global majority subjects are excluded, reified into markers of ethnicity, or not collected at all. To put it another way, health differences between ‘different Latino sub-ethnicities’ are made indicative of racial difference at a group level, while differences between white subjects are hooks for personalising health profiles.

This difference shows how racialisation works to distinguish humans into groups that are defined by their environment and those who are defined by their pathology. At a wider level, this is one of the ways that the Global South and its migrant diasporas are fixed in broad-stroke epidemiological health contexts, while the Global North is a site of biomedical enhancement. These are two very different registers of evolutionary time, the first based on (sub)species survival and the second on transcending the limits of human biology in view of extending human life and its capacities further and further. Environmental racialisation is connected to logics of national belonging and the hygiene of the body politic, whereby bad environments are figured expendable and temporary, its residents both embedded in abnormal and deviant environments, and disconnected from true national soil.

## Fire, soil and living with the dead

Bernard’s *Surge* opens with an epigraph from the Book of Hebrews: ‘For here we have not an enduring city, but we are looking for the city to come’.49 The line carries a longer exhortation to persevere, to survive and to endure in the face of persecution. It invokes destruction as it points towards futures beyond a precarious encampment. This future begins outside the gates of the camp, where the bodies of animals whose blood has been used as sacrificial offering are burned. The line inscribes a vision of the social that does not stop at offering the blood of others as atonement for collective sins, because these sacrifices promise neither longevity nor stability. Responding to the gaps, omissions and traumas of black British archives as a response to those held in London’s George Padmore Institute, Bernard’s 2019 collection offers a plurality of influences for black British life, while resisting forms of authoritarianism that demand the blood of others. The poems hold the silences, occlusions and disappearances of these histories between two fatal fires: New Cross in 1981 and Grenfell Tower in 2017. Alongside the filed documentations of these histories, Bernard deploys lyric to conjure a continuing ‘we’ in search of a city to come, speculatively transforming death-worlds of the living dead into a praxis of living with the dead. The poems in *Surge* move through rhetorical figures of unfixing, making insensible the spatiotemporal grammars that make sense of the abandonment of bodies to trajectories of debility through racialised imaginaries of delay and deviance.

The subjects of Bernard’s ‘Chemical’ are the ghosts produced in the ‘now-gone’ of the Grenfell Tower fire, hovering between origin and trauma, burned building and ‘frayed spirit’.50 The poem begins in mid-air, an undetermined place in the sky where these spectres have been ‘dragged’:

And all of their ghosts are burning

above the city: Some fires burn

pink as damaged blossom.

‘Chemical’ begins halfway through a sentence, somewhere between the temporal realms of present and past continuous, in a happening (‘burning’) linked to previous happenings (‘some fires’) that continue to happen (‘burn’). In Gothic imaginaries, ghosts are often tied to their environments: a part of a house or a patch of landscape—wood, hill, river, the side of a road—appearing between trees as tricks of the light and disappearing with the time it takes to do a double-take. Not so for the ghosts in ‘Chemical’, who rise up in the darkness of the middle of the night, in flames, from the remnants of the here-nameless burning tower. Smoke makes indistinct and phantom shapes, ballooning and dispersing into a milieu of other urban pollutants; smog as haunting. This burning is specific, but there is a general context here too: ‘some fires’ intuits a longer history, a ‘damaged’ series of burning flesh (‘pink…blossom’).

Whose are these ghosts? They are the spectral forms of bodies that belonged to now-anonymous fragments of flesh, called into a plural time zone formed by a confluence of flammable toxins, organic matter and historical damage:

Those broken vessels, bruised, lit

and upward streaking, rose-hot capillaries

ignite the dead’s ragged cloth and unshrooms

them to gas.

This is a displacement of bodily metaphors onto the phenomena and effects of fire, accompanied by adjectives of corporeal vulnerability (‘broken’ and ‘bruised’). Vessels and capillaries that ensure the traffic of circulating blood shift to an image of fire streaking upwards. ‘Rose-hot capillaries’ match the pink of ‘damaged blossoms’, flesh and fire merging in the translucence of corporeal tones. The fire becomes an organism, an agent with the capacity to ‘ignite the dead’s ragged cloth’, some malevolent entity that consumes this impoverished scene. ‘Unshroom’ sounds like mushroom, a neologism whose sonic valence recalls the shapes of gargantuan explosions that take over horizons: the spectacle of atomic bombs, and the frozen, broken, contaminated bodies it leaves behind, resurrecting organic forms in obliterating guises.

Grenfell Tower began to burn just before 01:00 on 14 June 2017. When fires burn, they emit poisonous gases and particles across air, soil and water. Soon after the fire, residents of the tower block expressed concerns that while Public Health England had released data and advice on the air quality, smoke exposure, asbestos and clean-up process after the fire, there were no data being released on soil contamination, which posed the greater health risk for those who would continue to live in the area. While data on air pollution after the fire showed the aftermath to be low risk, no similar assurances had been made about the possibility of soil contamination, nor had advice been given about mitigating potential long-term environmental effects. Seventeen months after the fire, and in response to these concerns, a research team from the Centre for Fire and Hazards Sciences at the University of Central Lancashire led by Anna Stec undertook an independent study into contamination in the soil around the site. Stec’s team found significantly high levels of a number of toxicants, including evidence of polychlorinated dibenzo-p-dioxin concentrations ‘60 times greater than UK urban reference soil levels’, benzene levels ‘40 times greater’, and levels of polycyclic aromatic hydrocarbons ‘approximately 160 times greater’.51 The published report recommended further analysis around the tower ‘to understand possible health risks’.52 The soil around the tower was highly toxic and presented (and continues to present) various potential health risks to its remaining residents.

Before the report was published, its findings were reported in *The Guardian*, and the Ministry of Housing, Communities and Local Government focused on damage limitation. In March 2019, they set up a review of soil contamination levels that would involve community participation, feedback, and involve contributions from an independent science advisory group, including Stec. As in Galanter’s study, residents were asked to be experts in their place of residence, consulted about both the uses and aspects of land prior to the fire, as well as the design of the investigation itself, ‘to ensure that testing happens in the right places’.53 The figure of the local expert gestures to broader movements of citizen science, long-term efforts to democratise processes of research and policy-making in science, and to promote a feedback loop between corporate, governmental and civil society, while also a longstanding tactic of colonial statecraft. In April 2019, the Ministry announced that the investigation would be undertaken by an independent specialist contractor, AECOM, to carry out soil checks at Grenfell. The meeting notes from 11 April 2019 show that, after a review of Stec’s paper, the scientific advisory group concurred that ‘there was no reason to recommend changing current public health advice nor the approach to soil risk assessment’.54

It is not within the remit of this article to determine the validity or circumstances of this judgement. Stec’s paper drew attention to the question of health advice, and whether or not it was safe for the remaining residents of the Grenfell locality to continue to live next to it. At several points, the review process circumvents the question of governmental responsibility in both local and national governance, skirting the issue of substantive reparation beyond rehousing and financial compensation. Who is accountable, and for what? The failure to address these questions is made possible by putting the onus onto a mythological ‘local community’, imagined as a monolithic entity that regulates its parameters (who is included and who is not), has a clear sense of its short-term and long-term aims, and is able to demonstrate a level of expertise about the locality that will contribute to recommendations. While those who participated in the community workshops demonstrated these capacities, the purpose of the workshop was not to learn from them, but to engage in a publicised performance of active listening.

There is another powerful imaginary at work here: the correlation of soil and national belonging, ‘of the soil’ as a description of racialised citizenship. Many of the residents of Grenfell were not British citizens. As Ida Danewid notes, Grenfell Tower was ‘predominantly occupied by London’s racialised poor – by Nigerian cleaners, Somali carers, Moroccan drivers’.55 Making those remaining on the site responsible for knowing about common uses of land not only positions them as experts in their locality, but also fixes them to this locality. There are many remaining residents who might not be able nor want to participate in workshops connected to governmental agencies, as well as those who have not lived on the site for long enough to answer the topographical questions being asked in relation to possible long-term effects. The review’s process privileges a model of citizen feedback that ends up placing responsibility for adequate investigation (experimental design) back on those who are seeking redress: on the community who are experiencing the worst fallout from the fire to take partial responsibility for its aftermath. As well as this, it tacitly assigns a moral economy of citizenship—implicitly racialised—in which knowing the land becomes a condition of remaining on it and being cared for within it.

The unfolding of this process signals the way that the violence of a negligent, necropolitical and ‘racial structuring of neoliberal urban governance’56 is turned around on residents living in its fallout, who are positioned not only as responsible for the aftermath, but also fixed to the site. This stands out in the lip-service to community participation while contracting a private corporation to carry out soil checks, in the effective dismissal of the Stec group’s recommendations to review and revise public health advice, and in the possibility that future failures or omissions of the review will be shared with the community and billed as a failure of collective expertise. This is the compounding of environmental racism into environmental racialisation, tying the remaining residents of Grenfell to a toxic environment, where flammable walls have exploded across space into carcinogenic soil, a happening that continues into the present.

The poetics of influence in Bernard’s ‘Chemical’ move out of a logic of graphic fixing. Influences here are plural; the embedded body is as much a construct of imperceptible space as it is pressed, indefinitely, into the spectacle of the burning tower. Where do the dead go? This is the cipher to understanding the melancholic timbre of hermeneutic anxieties in the postgenomic era, with its interests in trauma and ancestry, contamination and heredity. What happens to the violent energies that bodies live through? Do they leave traces that bear marks in the present, in the form of recorded ancestral experiences: photographs and video reels, fragments of archives left in intergenerational codes? Invocations of influence, reparation and responsibility become ways of staying with the dead:

Screaming cackle. Frayed spirit,

unbecoming black we think makes up the unseen,

but that black is the last twisted shape

their bodies will take.

‘Chemical’ plays with the movement of colour through its organic and artificial compositions, using it to create invisible and undocumented images of the fire’s effects: the pink of a blossom standing in for the pink of burning flesh, rose-hot capillaries of flame signifying a body igniting and becoming fire. In this passage, ‘black’ moves from a becoming to a shadowed, spectral no-place—‘the unseen’—to the black of charred bodies. This undulation of meaning is at a distance from the typographical impulse of sovereign power, suggesting a place where sense is made in motion, and where this motion is a necessary tactic for safeguarding memory. Who has taken down the words of the dead? Their evidence has been burned up into a ‘screaming cackle’, a disembodied, undetermined, witch-like noise that arrives through the time zones of other fires and other burnings to witness this wreckage.

The word that stands out, positioned at the centre of the poem, and the only time it is used, is ‘bodies’. It is fixed at this formal heart by a convergence of colour—‘black’—and shape—‘twisted’: a derogatory racialisation that conjures deformity and monstrosity, and compounds racial identity with body type. The line should be an ending, a clause that holds ‘the last’ within it. The bodies are not positioned in the grammar of the past, but in the intuition of a future—‘bodies will take’. If the scene of death is an indication of what the future will inherit, then this ‘last twisted shape’ mimics a biological mutation, a ravaging of bodily architecture by chemical contamination that might be inherited by future generations. And yet, this is not an ending. The word is in negotiation with another word, ‘spirit’, source of the shifting ‘black’. Which realm gets to take the word with it: the bodies directed towards a future of ‘twisted shapes’, or the frayed spirit in motion, hovering between pink fire and black ‘unseen’? The poem goes on to plot the exploding structure:

The floor, the rooms,

liquid windows part absence, part gas.

Here, the architecture of the tower is broken down into parts—floor, rooms and windows—through its chaotic and seismic collapse. Windows radiating the heat of internal flames resemble the fluid motion of water, and ‘the floor’ arrives without warning in the middle of a line, as if the twisted bodies of the preceding sentence have fallen down upon it. This is an onslaught to rational configurations of space-time and to the arrangement of the built environment. The absence of a verb form leaves these parts of the building unfixed, without an action to bind them, loosed from each other and anything solid, now ‘part’ of another order of things and an uncalculated compound of ‘absence’ and ‘gas’. There is no sense of time, which has evaporated or gone up in flames. The sentence is like a photograph of a burning building, taken in haste and described and passed over as quickly as possible, in an avoidance of the terror of the sudden, abyssal absence it documents.

The bodies of the previous line are being unfixed from their environment. These lines effect a suspension of body from noise, of spirit from matter, and of the burning world from other worlds that lie proximate to it, ‘unseen’. It lays out a topography specific not only to the situation, but to the long moment of the fire and its proximity, through time, to other fires: New Cross, Haiti, Brixton. It configures its own environment where the infrastructure of the building is detached from any power of influence; some other negotiation is working between past, future and a timeless space of rest and peace—the negotiation over a legacy or inheritance, ‘the last’ thing to be passed on. It remakes the grammar of bodies attached to and fixed within their environment, loosening a host of other influences that, until now, have been closely compacted into the realm of the speculative and supernatural. These influences have been called out, recalling the viewpoint with which the poem began: ‘above the city’, watching from above, an alternative form of surveillance where spirits hover alongside drones.

Niewöhner and Margaret Lock have offered an alternative to the molecularised body as the central site of epigenetic investigation, which corresponds to this grammatical reconfiguration of environmental influence. Discussing the ambiguity around deciding what an influence is, they suggest instead reversing this model of environment influence → molecular effect, towards an understanding of the environment as that which is *made relevant*:

What constitutes “the environment” only becomes meaningfully defined in relation to a second entity to which that environment can be environment; or put in a different way – organism and environment always penetrate each other in several ways or co-construct each other.57

This extends process biology into the realm of pragmatics: ongoing signification that, for biosemiotics, is characteristic of life and its plural transformations. This does not just imagine objects relating *to* each other, and the local is not imagined something out there to be broken down into possible molecular referents; this is a poetics of ‘ceaseless relating’, where the local begins to move out of spatio-temporal fixity, losing coherence as a site of influence.58 In this formulation, the body is neither wholly universal not totally local, neither ‘of the soil’ nor reducible to any other human genome.

To expand this intuition through Édouard Glissant, it becomes possible to encounter 'identity as no longer completely within the root but also in Relation.'59 For Glissant, this is errantry without the 'melancholy and extroverted luxury of uprooting', where travel is no longer reducible to spatial reorientations but is 'a pleasurable, if privileged, time': 'the ontological obsession with knowledge here gives way to the enjoyment of a relation'.60 In the fantasy of attaching bodies to soil as a description of biological origin, soil is a naturalised border where necropolitical governance equates to forming domestic and highly surveilled ghettos—of detention centre, prison and housing estate, reinforced by systemic exclusion, while the sea is a site for making bodies disappear. The Grenfell fire brought these death-worlds together in a colossal manifestation of racialised violence which, as Danewid argues, demonstrates how:

the “making” of global cities often goes hand in hand with racialised policies and practices designed to “clean up the streets” through revitalisation programmes and plans to displace actually existing inhabitants, which are cast as deviant, criminal, and out of place.61

Environmental racialisation is a global strategy of city-making and a form of neocolonial settlement, insofar as the boundaries of the nation state are figured around geographical placeholders mapped onto racialised bodies. Key here is how these spaces are *produced as* deviant, unclean and unregulated, when in reality they are subject to much greater levels of state surveillance and ‘cleaning’ programmes, under the guise of welfare.

‘Chemical’ extends another direction to those who inhabit its infrastructure, figuring disruption as the long memory of bodies seized, stolen and obliterated, rather than abnormal or deviant types, and practices of making life livable in a plurality of elsewheres that traverse the living and the dead. After the loosening of bodies from architecture, another element arrives: wind, which ‘breathes sideways’ and scatters the soot of the ‘ghosts … now-gone’:

back to the cold air making its way towards

a darker past, the true past, there at spirit level.

These final lines are an act of planting in the absence of a fixed soil, across an indeterminate space. The wind is carrying material traces of the bodies further up the poem into ‘cold air’, but they are no longer bodies: they are ‘ghosts of the now-gone’. This ‘now-gone’ is a temporal compound that solders past and present in a fragile, awkward union, a way of holding together a painful affect of immediacy and loss—a sudden going, an unanticipated departure, a shock to chronological time and its undoing. The scattering of soot by the wind brings these ghosts back from ‘there’ to ‘here’, from an eternal no-place to the time of the now-gone. Their traces become part of a longer history, ‘a darker past, a true past’. The figure of a ‘true past’ plays on the lure of origins, which is transmitted from the realm of soil, body and habitation into another infrastructure, higher than flames, where air and time tangle into another atmospheric layer, ‘the unseen … at spirit level’.

## Conclusion

This article has described poetics of influence at work in postgenomic experimental imaginaries by focusing on ways that epigenetic studies participate in processes of environmental racialisation. I have offered three examples in these imaginaries that move across concepts of space, methodological decision-making and arranging bodies in time: embodied contexts, occlusion and hypervisibility, and delay and deviance. Bodies are fixed to environments as a way of producing a good/bad distinction banked in recourses to eugenic determinations of hereditary value, *a priori* racialised, which allows the retention of biovalue in white environments. Central to this is the mobilisation of spatiotemporal embeddedness: differentiating epigenetic events and their respective communities through ancestral, geographical and physiological contexts. This graphic precision makes other configurations of space-time disappear. With this in mind, the article reads Bernard’s ‘Chemical’ as a working-through of these overdeterminations and their creation of death-worlds: what influence do the dead have over the living, not just in the form of inherited code but as a panoply of summonable influences? In the way it reconfigures the poetics of a necropolitical present into agonising and opaque compounds of effect, keeping causality superstructural, Bernard’s poem offers a vision of resurrection as reparation. The postgenomic era is still working out its questions about influences of being and being made human; my interest here has been to attend to its poetics of individuation and embodiment, for the sake of remembering demonstrations of collective life.

## Data Availability

No data are available.

## Notes

1. Achille Mbembe (2003), “Necropolitics,” Translated by Libby Meintjes, *Public Culture* 15, no. 1: 40

2. Alfred North Whitehead (1997), *Science and the Modern World* (London: Free Press), 199–200.

3. Jenny Reardon (2017), *The Postgenomic Condition: Ethics, Justice, and Knowledge After the Genome* (Chicago: Chicago University Press).

4. Reardon, *The Postgenomic Condition*, 174.

5. See Kaushik Sunder Rajan (2006), *Biocapital: The Constitution of Postgenomic Life* (Durham, NC: Duke University Press).

6. Isabelle Stengers (2014), *Thinking With Whitehead: A Wild and Free Creation of Concepts* (Cambridge, Mass.: Harvard University Press), 129.

7. Raymond Williams (1977), *Marxism and Literature* (Oxford: Oxford University Press), 129.

8. Williams, *Marxism and Literature*, 132.

9. See Clare Hanson (2020), *Genetics and the Literary Imagination* (Oxford: Oxford University Press); Catherine Malabou (2016), *Epigenesis and Rationality,* trans. Carolyn Shread (Oxford: Wiley); Wendy Wheeler (2006), *The Whole Creature: Complexity, Biosemiotics and the Evolution of Culture* (London: Lawrence and Wishart); Wendy Wheeler (2016), *Expecting the Earth: Life, Culture, Biosemiotics* (London: Lawrence and Wishart); Jesper Hoffmeyer (2008), *Biosemiotics: An Examination Into the Signs of Life and the Life of Signs*, trans. Jesper Hoffmeyer and Donald Favareau (Scranton, PA: University of Scranton Press).

10. E. P. Thompson (1967), “Time, Work-Discipline, and Industrial Capitalism,” *Past and Present* 38: 60.

11. Wendy Wheeler, “In Other Tongues: Ecologies of Meaning and Loss”, *Modern Forms*, https://modernforms.org/blog/colourful-speculation/

12. Megan Warin, Emma Kowal, and Maurizio Meloni (2020), “Indigenous Knowledge in a Postgenomic Landscape: The Politics of Epigenetic Hope and Reparation in Australia,” *Science, Technology & Human Values* 45, no. 1: 91.

13. Warin et al., “Indigenous Knowledge in a Postgenomic Landscape,’ 91.

14. G. C Spivak (1990), “Strategy, Identity, Writing,” in *The Postcolonial Critic: Interviews, Strategies, Dialogues*, ed. S. Harasym (London: Routledge), 35–49.

15. Thomas Wilson and Clarence Grim (1991), “Biohistory of Slavery and Blood Pressure Differences in Blacks Today. A Hypothesis,“ *Hypertension* 171, no. 1: 122–8.

16. See Nader Perroud et al. (2014), “The Tutsi Genocide and Transgenerational Transmission of Maternal Stress: Epigenetics and Biology of the HPA Axis,” *The World Journal of Biological Psychiatry* 15: 334–5; Rachel Yehuda and Amy Lehrner (2018), “Intergenerational Transmission of Trauma Effects: Putative Role of Epigenetic Mechanisms,” *World Psychiatry* 17, no. 3: 243–57.

17. Barbara Prainsack (2017), *Personalized Medicine* (New York, NY: New York University Press, 2017), 173.

18. Angela Saini (2020), “Stereotype Threat,” *The Lancet,* 395, no. 10237 (May 23): 1604.

19. Josie Gill (2020), *Biofictions: Race, Genetics and the Contemporary Novel* (London: Bloomsbury), 122.

20. Gill, *Biofictions*, 126, emphasis mine.

21. Stengers, *Thinking With Whitehead*, 130.

22. Frances A Champagne (2008), “Epigenetic Mechanisms and the Transgenerational Effects of Maternal Care,” *Front Neuroendocrinology* 29, no. 3: 386–97.

23. Sarah Richardson (2015), “Maternal Bodies in the Postgenomic Order: Gender and the Explanatory Landscape of Epigenetics,” in *Postgenomics: Perspectives on Biology after the Genome*,” ed. S. Richardson and H. Stevens, 21.

24. Richardson, “Maternal Bodies in the Postgenomic Order,” 221.

25. Stephanie Lloyd and Eugene Raikhel (2018), “‘It Was There All Along’: Situated Uncertainty and the Politics of Publication in Environmental Epigenetics,” *BioSocieties* 13, no. 4: 737.

26. Richardson, “Maternal Bodies in the Postgenomic Order,” 224.

27. Ibid., 224.

28. Ibid., 225.

29. Jörg Niewöhner (2011), “Epigenetics: Embedded Bodies and the Molecularisation of Biography and Milieu,” *BioSocieties* 6, no. 3: 288.

30. Richardson, “Maternal Bodies in the Postgenomic Order,” 227, emphasis mine.

31. S. Shostak and M. Moinester (2015), “The Missing Piece of the Puzzle? Measuring the Environment in the Postgenomic Moment,” in *Postgenomics*, ed. S. Richardson and H. Stevens (Durham, NC: Duke University), 203.

32. Becky Mansfield and Julie Guthman (2015), “Epigenetic Life: Biological Plasticity, Abnormality, and New configurations of Race and Reproduction,” *Cultural Geographies* 22, no. 1: 8.

33. Mansfield and Guthman, “Epigenetic Life,” 8.

34. Ibid., 9.

35. ibid., 10.

36. Jasbir Puar (2017), *The Right to Maim: Debility, Capacity, Disability* (Durham, N.C.: Duke University Press, 2017), xvi.

37. See Sut Jhally and Stuart Hall (2007), *Race: The Floating Signifier* (Northampton, MA: Media Education Foundation).

38. Anne Catherine Viuff et al. (2018), “Maternal Depression during Pregnancy and Cord Blood DNA Methylation: Findings From the Avon Longitudinal Study of Parents and Children,” *Translational Psychiatry* 8, no. 244 (November): 2.

39. Joshua M. Galanter et al. (2017), “Differential Methylation between Ethnic Sub-Groups Reflects the Effect of Genetic Ancestry and Environmental Exposures,” *eLife* 6: e20532, accessed October 4, 2020, https://www.ncbi.nlm.nih.gov/pmc/articles/PMC5207770/

40. Gill, *Biofictions*, 105.

41. See K Kamps (2001), “Environmental Racism, Tribal Sovereignty and Nuclear Waste,” *NIRS.org* (February), accessed October 3, 2020, https://www.nirs.org/private-fuel-storages-proposed-interim-high-level-nuclear-waste-dumpsite-on-the-reservation-of-skull-valley-goshute-tribe-40-miles-west/

42. Achille Mbembe (2019), *Necropolitics* (Durham, NC: Duke University Press).

43. Cheryl Teelucksingh (2007), “Environmental Racialization: Linking Racialization to the Environment in Canada,” *Local Environment: The International Journal of Justice and Sustainability* 12, no. 6: 645.

44. Teelucksingh, “Environmental Racialization, 645.

45. Mel Chen (2015), “Unpacking Intoxication, Racializing Disability,” *Critical Medical Humanities* 4: 27.

46. Chen, “Unpacking Intoxication,” 27.

47. See Walter Rodney (1972), *How Europe Underdeveloped Africa* (London: Bogle-L’Ouverture Publications).

48. Chen, “Unpacking Intoxication,” 27.

49. Hebrews 13:14, *The Holy Bible*, *New International Version* (Grand Rapids: Zondervan Publishing House, 1984).

50. Jay Bernard (2019), “Chemical,” *Surge* (London: Chatto & Windus), 47.

51. Anna A. Stec et al. (2019), “Environmental Contamination Following the Grenfell Tower Fire,” *Chemosphere* 226: 576.

52. Stec et al. (2019), “Environmental Contamination Following the Grenfell Tower Fire,” 576.

53. Ministry of Housing, Communities and Local Government (2019), “Grenfell Environmental Checks – Feedback From the Community Workshops and How Your Input Will Be Taken Forward,” *Gov.uk* (May 28), https://www.gov.uk/government/publications/grenfell-environmental-checks-feedback-from-community-workshops

54. Grenfell Science Advisory Group (2019), “Advice to Grenfell Tower Fire Ministerial Recovery Group, 4 March 2019: Minutes of Meeting #4 11 April 2019,” *Gov.uk* (April 11), https://assets.publishing.service.gov.uk/government/uploads/system/uploads/attachment_data/file/806702/science_advisory_meeting_minutes_meeting_4.pdf

55. Ida Danewid (2020), “The Fire This Time: Grenfell, Racial Capitalism and the Urbanisation of Empire,” *European Journal of International Relations* 26, no. 1: 292.

56. Danewid, “The Fire This Time,” 303.

57. Jörg Niewöhner and Margaret Lock (2018), “Situating Local Biologies: Anthropological Perspectives on Environment/Human Entanglements,” *BioSocieties* 13: 690.

58. Niewöhner and Lock, “Situating Local Biologies,” 681, 690.

59. Édouard Glissant, *Poetics of Relation* tr. Betsy Wing (Ann Arbor, MI: University of Michigan Press, 1997), 18.

60. Glissant, *Poetics*, 19.

61. Danewid, “The Fire This Time,” 291.
